# Different perspectives of immunizations during pregnancy

**DOI:** 10.3906/sag-1910-23

**Published:** 2020-04-09

**Authors:** Gökce CELEP, Aysu DUYAN ÇAMURDAN, Fatma Nur BARAN AKSAKAL, Osman Fadıl KARA

**Affiliations:** 1 Department of Pediatrics, Faculty of Medicine, Amasya University,Sabuncuoğlu Şerefeddin Education and Research Hospital, Amasya Turkey; 2 Department of Pediatrics, Faculty of Medicine, Gazi University, Ankara Turkey; 3 Department of Public Health, Faculty of Medicine, Gazi University, Ankara Turkey; 4 Department of Obstetrics and Gynecology, Medical Faculty, Amasya University,Sabuncuoğlu Şerefeddin Education and Research Hospital, Amasya Turkey

**Keywords:** Immunization, pregnancy, influenza, adult type pertussis vaccine (Tdap), postexposure prophylaxis

## Abstract

**Background/aim:**

Pregnant women and infants are at risk of severe lower respiratory tract infections induced by influenza or pertussis. The uptake of both vaccines is poor in spite of proven benefits and safety. We aimed to determine the knowledge and attitude of pregnant women and their primary healthcare providers towards immunization during pregnancy.

**Materials and methods:**

This cross-sectional and interventional study was conducted in northern Turkey in 2016. It had 3 different groups including 786 pregnant women, 146 primary healthcare staff, and 97 family practitioners. Different questionnaires were filled by each group. The intervention phase of the study consisted of education of the expectant mothers about immunizations during pregnancy.

**Results:**

786 pregnant women aged between 17–44 years were enrolled to the study. Most of the participants had favourable attitude about vaccination, but only 1.1% had influenza immunization, none had Tdap immunization. None of the participants joining the intervention stage were immunized. The healthcare staff and family physicians had knowledge about vaccinations, but had abstention for administration. Postexposure prophylaxis was also provided by referral centres.

**Conclusions:**

Most of the participants either pregnant women or healthcare workers were not vaccinated against pertussis and influenza. Dissemination of maternal immunization must be supported by the team work of healthcare professionals, authorities, universities, professional associations, stake holders, media and patients with current, evidence based knowledge.

## 1. Introduction

Women at childbearing age are typically immunized against vaccine preventable diseases (VPD) through vaccination or disease exposure [1,2]. However, immunity may decrease over time and pregnant women or new-borns become susceptible to VPD with complications. [1,3]. Ideally, all women should be immunized before pregnancy, but approximately 25% of pregnancies are unplanned [4]. Maternal immunization provides protection for the mother and baby until the primary immunization schedule initiates [5]. Live attenuated vaccines are contraindicated in pregnancy. Most inactive vaccines are known to be safe and beneficial for maternal and foetal health [1]. Maternal and neonatal tetanus has been eradicated in many countries (including Turkey) by tetanus-diphtheria (Td) vaccination during pregnancy [6]. This experience has been considered as an opportunity for other VPD. There are increasing data showing maternal immunization with inactive influenza (IIV) and adult type tetanus, diphtheria, and acellular pertussis vaccine (Tdap) is effective and safe to reduce severe respiratory tract infections (RTI) [7,8]. Tdap and IIV are recommended to pregnant women during every pregnancy, influenza at any gestation age (preferably in the second trimester), and Tdap during the third trimester for optimal transmission of the maternal antibodies [7,9–12]. The expectant mother is also protected from the disease, which may be more complicated than nonpregnant peers. This provides a “cocoon” around the infant during confinement. The uptake of both vaccines is poor in spite of proven benefits. Both pregnant women and their healthcare providers are reluctant [5]. The Turkish Republic Ministry of Health (TRMH) recommends routine Td and influenza vaccinations for pregnant women according to adult vaccination schedules [13]. In Turkey, obstetrical follow up is provided by obstetricians, family physicians, and midwives in different healthcare settings. Vaccinations are under the control of primary healthcare centres [13]. During pregnancy 97% of women receive antenatal care from a healthcare provider at least once [4]. Therefore, pregnancy becomes an opportunity for immunization.

In this study, we aimed to determine the knowledge and attitude of pregnant women and their primary healthcare providers towards immunization during pregnancy in northern Anatolia. Also, we aimed to identify the factors that affect the eventual decision on immunization. To determine the potential barriers that could be addressed for improving maternal immunization coverage, this cross sectional, descriptive, and interventional study was performed.

## 2. Materials and methods

The survey phase of the study was conducted in the obstetrics outpatient clinics of a tertiary healthcare centre between June 1st and December 31st 2016. Since the flu season is between November and April in our country, the duration of the study was planned to cover the flu and vaccination season [14]. A questionnaire was prepared by reviewing the literature for all pregnant women applying to pregnancy follow-up unit. The questions were about socio-demographic features (age, residence, educational level, working status, occupation, and monthly income), obstetric and medical history. General attitudes about immunization, vaccination status at pregnancy, sources of information, attitudes towards immunization during pregnancy and general influenza vaccination status, knowledge, and experience were documented. Reasons for getting/not getting vaccinated during pregnancy were also asked. The staff of the unit was educated about the survey. The applicability of the questionnaire was tested with 20 pregnant women. Written and signed consent forms of each participant were taken prior to administration. Questionnaires were filled out with the help of staff through face-to-face interviews lasting approximately 10 min. 

In order to educate women about pregnancy and baby care, childbirth education class were offered in our hospital. The “intervention” phase of the study was designed within this context. In addition to routine pregnancy immunizations, a new course about influenza and Tdap was added to the content, emphasizing that these vaccines can be safely administered during pregnancy. At the end of the study, participants of the course and survey were called by telephone and asked whether they had influenza and/or Tdap vaccines.

Assistant healthcare staff, midwives, nurses, and health officials carrying out pregnancy follow-up service in primary healthcare centres participated in the study. They filled in the questionnaires via e-mail and telephone calls. Age, gender, working place, and active occupational time in the profession were asked. The number of pregnant women was followed up, the rate of follow up compliance, Td vaccination rate, acceptance and rejection rates of vaccination and reasons, adverse events after immunization, and personal thoughts about immunization during pregnancy were questioned. Their knowledge about vaccines and routine administration (influenza, Tdap), and pregnancy immunization history of their own or their partners’ were questioned.

Family physicians that carried out pregnancy follow-up service in primary healthcare centres also participated. In the first part of the survey, information about age, gender, work place, active working time in the profession, number and pregnancy follow-up rate, Td immunization rate, acceptance/rejection rates, nonroutine influenza and Tdap vaccines justification, reasons for recommending/not recommending, expert referral rate for immunization, their immunization history during pregnancy, exposure to vaccine preventable complications in pregnant women, and their competence in vaccination were asked. In the second part, physicians were asked to solve the case scenarios about immunization during pregnancy when indicated. Seven cases and 11 questions were scored as 1 point for every correct answer. 

### 2.1. Ethics

The study was approved by the ethical committee of a university with the decision number: 77082166-604.01.02.

### 2.2. Statistical analysis

Statistical analysis was performed using SPSS v15.0 (SPSS Inc,, Chicago, IL, USA). The continuous variables were investigated using visual/analytical methods. Descriptive statistics were presented as frequencies, percentages, arithmetical mean ± standard deviation, and median (minimum, maximum). Categorical variables were compared using Pearson’s chi-square, Yate’s corrected chi-square, and Fisher’s exact test where appropriate. Mann Whitney-U test was used for the comparison of the 2 groups when the data were not normally distributed. A P-value of less than 0.05 was considered as statistically significant.

## 3. Results

A total of 786 pregnant women were enrolled in the study. Their ages varied from 17 to 44 (mean: 26.83 ± 5.23) years and the education level of 77.4% (n = 608) was at least secondary level. Most of them were housewives living at provincial area (n = 681, 86.6%; n = 406, 52.2% respectively). Approximately 70% (n = 538) of the participants were at the third trimester during the study and 78.1% (n = 614) were followed up by an obstetrician. 

Td was administered to 77.4% (n = 608) of the participants. Forty five (5.7%) participants avoided this routine vaccine without a reason and 121 (15.5%) pregnant women were in the early weeks of the first trimester to get vaccinated. Fifty-five of the participants (10.8%) were recommended influenza vaccination during pregnancy and the source of recommendation was primary health-care services in 47 (85.5%) patients. However only 8 (1.1%) of them had influenza vaccination. Eleven participants (1.5%) were recommended Tdap, but none of them got vaccinated. The main reasons for not getting vaccinated with influenza and Tdap during pregnancy were not having sufficient knowledge about these immunizations or not believing in their necessity. 

Most of the participants had a favourable attitude about vaccination in general and 57.5% (n = 449) thought that the vaccines were necessary and beneficial for health. Thirty-one (3.9%) pregnant women believed that vaccines were unnecessary and unsafe. However, 31.6% (n = 248) pregnant women had no sufficient general knowledge about vaccines. Immunization during pregnancy was a novel title which should be consulted with the attendee physician. Most of the participants (n = 437; 63.5%) stated that they would get vaccinated if their physician offered, but 11.6% (n = 91) of them would never get vaccinated during pregnancy. When the knowledge about the effect of vaccines protecting the infants from severe respiratory tract infections was shared, 81.4% (n = 637) of the expectant mothers stated that they would get vaccinated.

In the “intervention phase” of the study, 142 participants joining both the survey and childbirth education class were called asked about immunization with influenza and/or Tdap by phone. None of the participants got vaccinated as their obstetricians did not recommend (Figure). 

A total of 146 healthcare workers aged between 22 and 66 (mean: 34.26 ± 7.04) years old participated in the survey. Nine were men and 24% (n = 35) was employed at the provincial centre. The median occupational time was 10 years (1–40). 

**Figure 1:**
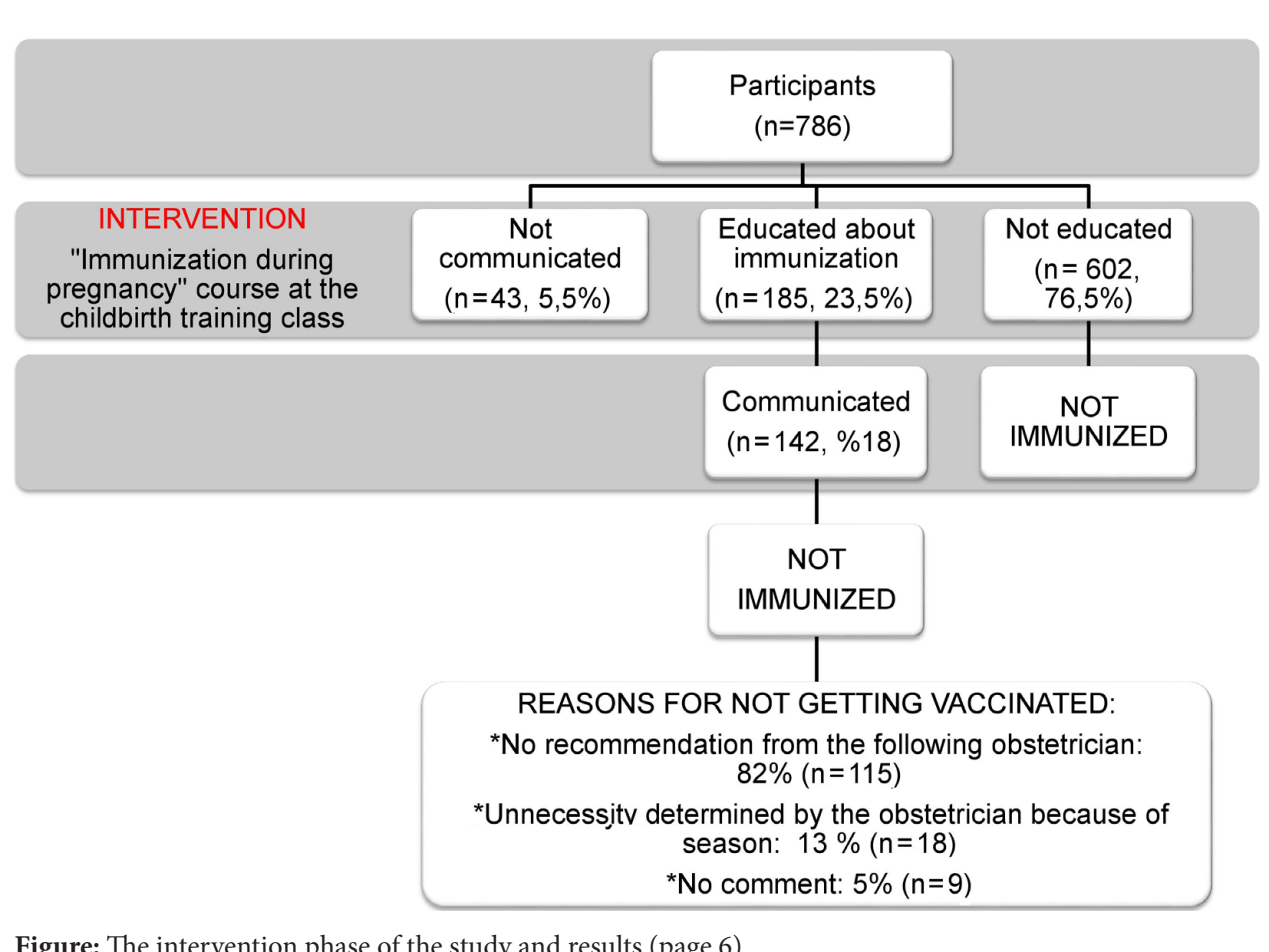
The intervention phase of the study and results (page 6).

Encountering antivaccination attitude during pregnancy was reported in the 24.7% of the study group. Most common reasons were anxiety about side effects, obstetricians’ statements about immunization, mistrust to vaccines, personal belief in needlessness, pain/injection fright, lack of knowledge, having been already vaccinated, religions and medical reasons. However, pregnant women religions persuaded by family physicians at prenatal visits, provided Td vaccination rate as 94%. Only 4 (2.7%) of the participants reported administration of hepatitis B and influenza vaccines. One hundred and sixteen professionals had pregnancy history and 105 (92.9%) were vaccinated with Td, but only one had influenza immunization. Two midwives experienced vaccine preventable complications during pregnancy. Both cases were influenza complicated with severe lower RTI. Age, gender, occupational time, and work place had no statistical significance on attitudes about non-Td (influenza, Tdap) vaccinations during pregnancy (P > 0.05). 

Thirty-five women (36.1%) and 62 men (63.9%) from a total of 97 family physicians aged between 25 and 61 years (mean 39.70 ± 7.70; median: 40) participated in the study. The number of pregnant women followed up was 4–45 (median 21), all attended prenatal visits at least once. The rate of Td vaccination was 98%. In practice, 35% of the practitioners reported meeting vaccination opposition. However, the final decision was the acceptance of Td vaccination following the official recommendation of TRMH. When asked about their own attitudes towards non-Td immunizations, 43.3% (n = 42) reported recommendation, 94% of these was influenza, 37.5% Hepatitis B, and 14% Tdap. History of chronic illnesses was an important factor (69.1%; n = 67). However, several reasons for reluctance were also determined including lack of routine practice, scepticism about necessity, worry about side effects, and lack of education, desire to consult with an expert, problem with risks, and those who were against vaccination. Four of the physicians reported influenza immunization during pregnancy. Socio-demographic characteristics or immunization history had no effect on non-Td vaccine recommendation (gender: P = 0.32; age: P = 0.76; working place: P = 0.59; immunization history: P = 0.09). 

For postexposure prophylaxis or medical indication, 35.2% (n = 32) of physicians recommended vaccination to pregnant women. In addition, 40.6% (n = 13) of these physicians also recommended non-Td vaccines, but it was not statistically significant. Needing expert consultation for these administrations (53.7%; n = 29), reluctance to take responsibility (16.7%; n = 9), complication follow-up anxiety (13%; n = 7), and patient preference (7.4%; n = 4) were reasons of referral to the advanced centre.

The mean score of physicians about case scenarios was 6.69 ± 0.26 points (out of 11 points) with a median of 7.00 (3–11; min-max). The 62.5% (n = 34) of the physicians who scored above the average also recommended immunization in case of medical necessity and non-Td vaccines during pregnancy (Table). 

**Table:** The summary of case scenarios, management, and rate of family physicians’ correct answers (page 9) [1].

Case summary	Immunization	Correct answer,rate of correct answer (%)
A pregnant who needed to undergo splenectomy after acute trauma	Haemophilus influenza type b (Hib)	Yes,49 % (n = 47)
	Polysaccharide pneumococcal vaccine	Yes,56.7% (n = 55)	Quadrivalent conjugated Meningococcal vaccine (Men ACWY)*	Yes,44.3% (n = 43)
	Immunization of a pregnant woman planning to pilgrimage	Quadrivalent conjugated Meningococcal vaccine (Men ACWY)	Yes,46.4% (n = 45)
Postexposure prophylaxis for rabies after a dog bite	Rabies vaccine	Yes,78.4% (n = 76)
Serology negative pregnant woman’s susceptible exposure to chickenpox	Varicella vaccination	No **79.2% (n = 76)
Rubella immunization consultation of a serology negative pregnant woman to prevent congenital rubella syndrome	Rubella vaccination	No **74.7 % (n =71)
Improper vaccination hepatitis B schedule of a nurse	Hepatitis B immunization	Yes 65.6 % (n = 63)
Hepatitis A vaccination of a seronegative pregnant woman	Hepatitis A immunization	No ***%38.9 (n = 37)
Poliomyelitis immunization of pregnant woman whose vaccination schedule is incomplete for polio and obliged tovisit Afghanistan as a United Nations officer	Inactive poliomyelitis vaccine	Yes %57 (n = 53)
	Oral polio vaccine	No58.5% (n = 55)

*no data is available for MenB vaccines; the situation should be evaluated individually. **should be postponed to postpartum period. ***should be postponed to postpartum period if there is no risk factors, susceptible exposure or an epidemic.

## 4. Discussion

This study was designed to determine the attitude of pregnant women and their primary healthcare providers about immunizations during pregnancy. Of the 786 pregnant participants, 94% had Td, 1% had seasonal influenza vaccine, and none had Tdap. Knowledge of pregnant women about the process was insufficient and the most important determinant of acceptance was the recommendation of their attendee physician. Very few of the healthcare personnel administered non-Td vaccines. Although physicians had knowledge about immunizations during pregnancy, they usually administered these vaccines with expert consultation. The obstacles in the field were the lack of information, the myths on side-effects and the statements of obstetricians about immunization. In this study, 50% of the pregnant women getting influenza immunization were recommended by the family physician and only 10% by obstetricians. Also, vaccination rejection was prevented when immunization was officially recommended or had been administered previously. Thus, Td vaccination rates were above 90%, whereas the rate of influenza vaccination was only 1%. Although the risk class for teratogenicity of influenza and Tdap is C, the awareness and acceptance rates were low among pregnant women and healthcare workers. This result suggests that habits rather than evidence based data are more effective on attitude and behaviour development.

General attitude about vaccines is particularly important in receiving immunization during pregnancy. The most common reasons of rejection were insufficient knowledge about vaccines, concerns about vaccine safety and adverse effects, mistrust against vaccine efficacy, and underestimation of disease risk [15–18]. Lack of knowledge about immunizations during pregnancy seemed to be the most important issue. The highest rate of influenza immunization in Turkey during pregnancy was in the 2009–2010 pandemic influenza season (9.1%), which was much lower than USA (45.7%). One reason for this high rate may be due to the TRMH recommended vaccination after the 20th gestational week [19,20]. Influenza immunization rates are under desired levels in developed countries. In a study conducted within the European Union, vaccination rate in pregnant women in 2011/12 was reported as 2.0% in Slovenia and 30% in England. In United States, this rate was 47% [16,21,22]. Differences between countries may be related to different information, communication techniques, formal vaccination schemes, insurance coverage, and/or general vaccination attitudes. Also, the earlier the influenza vaccine was added to the pregnancy immunization schedule, the higher the rate of vaccination would be. It has been administered in the US since 1967; therefore, the vaccination rate is higher [23]. The formal recommendation was recently published by the TRMH [24]. 

In this study, 8 of 786 pregnant women had an influenza vaccine. The most common reasons for not getting vaccinated was the lack of knowledge about the vaccine (89.2%), thinking vaccination as unnecessary, mistrust, unwillingness, and concerns about safety. However, 63.5% of the participants declared that they would accept vaccination if their physician recommended. When the protective effect of immunization for the baby was declared, the acceptance rate raised to 81.4%, which is consistent with the literature [5,19]. Although living in a city was a facilitating factor, it was striking that none of the vaccinated women of this study lived in the city [17,25]. All of them had secondary or higher grade education supporting that vaccination rates were increased when education level increased [19,20,26]. 

In the intervention phase of the study, a lecture providing awareness for the participants was given about immunization during pregnancy. However, since the lecture did not come from their physician, none received the vaccine. Informing pregnant women and pointing baby’s health through physicians are important variables for increasing vaccination [27]. 

Lack of knowledge about immunization during pregnancy is typically uncommon in developed countries. Several reasons for vaccine rejection in those studies study include; pre-existing treatment options already available for influenza, no serious disease threat, vaccination causing flu, trypanophobia, mother/baby concern, lack of trust in healthcare and vaccine efficacy, media bias, lack of information vaccine locations, and financial burden. Strategies to increase vaccination consent include; education, make certain inter-sectored support, allotted time for caregivers to educate patients on vaccinations during visit, make increased vaccines accessibility, disseminate education/propaganda on vaccinations, enhance reliability of sources for dissemination, alter legislation, and increase methods for side-effect reduction [5,28]. Although the general vaccination and Td rates are high, 27.4% of the healthcare personnel reported rejection due to the same reasons listed above. A lot of physicians (43%) recommended nonroutine vaccines to pregnant women. However, the rate was lower than developed countries and the most common recommended non-Td vaccine was influenza. Presence of chronic disease, history of influenza vaccination during pregnancy by physician, the belief in the cocooning strategy, and knowledge about vaccinations were factors increasing the vaccination recommendation rate. The reasons for not offering non-Td immunizations were also reported and included; religious beliefs, scepticism of vaccinations, concerns about baby’s health following vaccination, lack of proper education about pregnancy and vaccinations, and expecting the requisition from the patient. This problem can be solved by official application formulas from the ministry, as in the case of Belgium and Canada [26,29]. Epidemics or dramatic events like in 2009 change the attitude of people.

In general, immunization seems to be the responsibility of primary care services. In Spain, pregnancy follow-up is usually provided by the primary care units. Midwives are the main source of immunization information. Influenza immunization rate was reported as 40.5% [29,30]. In this study, the main source of recommendation was primary caregivers rather than obstetricians. Studies in the literature focus on the abstention of gynaecologists in practice and guidance rather than lack of knowledge. We obtained the data about obstetricians’ attitudes indirectly as the study was designed for primary caregivers, which is a limitation of this study. The literature data and the statements of the medical staff overlap; there is a lot of information pollution and abstention caused by traditionalist tendency [29]. Tdap has been recommended during pregnancy in developed countries recently. In this study, none of the participants got Tdap. The awareness of assistant healthcare personnel was low (3.4%) and recommendation rate of physicians was 14% (n = 6). Tdap is a new vaccine in Turkey. False beliefs that history of whooping cough and/or immunization during childhood provide permanent immunization causing no vaccination coverage. Tdap in pregnancy has not been studied in Turkey. However, a survey conducted in Belgium in 2014–2015 showed that the rate of Tdap immunization was 65%. Interestingly, 82.4% of this was recommended by family physicians. The most important factor facilitating acceptance was the expectant mother’s education level [26]. Since 2004, Immunization Advisory Committee in Germany has recommended immunization to all women of childbearing age, to all adults who may be in close contact with babies, and those that have not been vaccinated with Tdap in the last 10 years (cocooning strategy). Tdap is offered before pregnancy or during postpartum period, whereas it is routine in England and USA during pregnancy. The immunization rate was high in England, but low (1.7%) in Italy [31,32]. Cocooning strategy is supported, but it is difficult to apply in practice. Therefore, it cannot replace, but support maternal immunization [33,34]. 

Postexposure prophylaxis is also possible for pregnant women. This problem seems to be solved by infectious diseases experts in referral healthcare centres. In this study, 56.7% of physicians recommended and administered post exposure prophylaxis during pregnancy. No studies evaluating the knowledge and attitude of the physicians’ on postexposure prophylaxis have been found in the literature.

Immunization during pregnancy is beneficial and safe for the mother and the baby. In spite of all evidence-based benefits, the process creates hesitation for pregnant women and healthcare providers. Td rates are above 90%, but influenza and Tdap during pregnancy are much lower than expected both in our province and in Turkey. The reasons for this problem are the lack of current and evidence-based information, the fact that obstetricians do not recommend immunization, and insufficient time for explaining vaccines to patients. Education and encouragement of physicians with current information can contribute positively to the process. A common thought is that distribution of information by obstetricians would assist in consent. Alternative protective methods for immunization are not as effective as vaccination for clinical protection, feasibility, and cost effectiveness. Cocooning via herd immunity is protective for the woman/child but, it has several limitations. 

Education and administration of vaccinations during pregnancy need to be implemented and funded during follow-up visits. Support for these programs should be encouraged by our institutions, caregivers, and legislators. Financial support by social security services will be important for distributing information about immunizations during pregnancy. 

A single dose of influenza vaccine should be recommended to all women planning pregnancy and for those expecting in their second trimester during the influenza season. Also Td dose closest to birth should be given as Tdap to protect the infant from pertussis related lower RTI. The common outcome of ​​all studies is that healthcare providers should recommend vaccination for the acceptance of pregnant women.

**Acknowledgements**

We would like to acknowledge the staff of pregnancy follow up unit for their contribution to data collection. Also we thank to all participants for filling the questionnaires willingly. The study was approved by the Ethical Committee of Gazi University with the decision number: 77082166-604.01.02.

**Conflict of interest**

The authors declare no conflict of interest.
